# Spatial macrophage-fibroblast interactions in osteoarthritis synovium: inflammatory and fibrotic niches

**DOI:** 10.3389/fimmu.2026.1990411

**Published:** 2026-10-16

**Authors:** Huacheng Wei, Ming Lei, Chendeng Lao, Ronghe Gu

**Affiliations:** Department of Orthopedic Surgery, The First People’s Hospital of Nanning, The Fifth Affiliated Hospital of Guangxi Medical University, Nanning, Guangxi, China

**Keywords:** fibroblasts, macrophages, osteoarthritis, spatial transcriptomics, synovial fibrosis, synovium

## Abstract

Synovial inflammation and fibrosis often coexist in osteoarthritis, but a transition from one to the other has not been established. This Mini Review examines macrophage-fibroblast interactions in the surface lining, perivascular regions, and collagen-rich synovium. We integrate human spatial and single-cell studies with functional experiments and map reported cell states, markers, locations, and functions. Experimental evidence is clearest for inflammatory amplification. Signals from recruited macrophages can increase fibroblast chemokine and cartilage-degrading enzyme production, while fibroblast secretions and extracellular vesicles influence macrophage recruitment and activation. Oncostatin M can induce inflammatory responses without increasing collagen deposition. Matrix synthesis and mechanical responses characterize fibrotic niches, which may also contain fibroblasts with immunoregulatory functions. These findings favor a model of potentially coexisting niches with overlapping but partly separable inflammatory, matrix-remodeling, and tissue-maintenance functions. Spatial proximity in human tissue identifies candidate signaling partners; it cannot establish their dependence on specific signals or an inflammation-to-fibrosis sequence. We discuss experiments that link defined cellular sources to local responses and measure pain, collagen deposition, clearance of dying cells, and lubrication separately.

## 1 Introduction

The synovial lining supplies joint lubricants and regulates the interface between the joint cavity and tissue. Loss of proteoglycan 4 (PRG4, also known as lubricin) causes synovial overgrowth and cartilage damage in mice (1). In osteoarthritis (OA), lining hyperplasia accompanies inflammatory infiltration and matrix accumulation in the sublining (2). Synovitis is associated with knee pain and subsequent structural disease, although this relationship varies among patients (3, 4). Fibrosis also changes tissue mechanics through thickening and altered matrix organization (5, 6).

Both macrophages and fibroblasts include populations with distinct functions (7, 8), which complicates the interpretation of depletion experiments. In obese macrophage Fas-induced apoptosis (MaFIA) mice, systemic macrophage depletion increased neutrophil and T-cell infiltration without reducing OA severity after meniscal destabilization (9). By contrast, depleting lining macrophages with clodronate before collagenase-induced OA reduced osteophyte formation by 84% at day 7 and 66% at day 14 (10). Differences in the site and timing of depletion, as well as the metabolic setting, must be considered when comparing these outcomes.

Macrophage-fibroblast crosstalk refers to signals through which either cell type changes the recruitment, activation, survival, or output of the other (11, 12). Its effects depend on the cells involved and their location. Single-cell sequencing identifies candidate partners, spatial assays locate them within anatomical niches, and ligand-receptor analysis suggests signals for functional testing (13). Here, we examine how these interactions amplify inflammation, remodel matrix, and preserve synovial function. We draw on human knee and hip OA and experimental OA, identifying rheumatoid arthritis (RA) evidence where it informs interpretation. We propose that inflammatory and fibrotic niches may coexist, without assuming that one inevitably develops into the other.

## 2 Spatial architecture and cellular states

### 2.1 Homeostatic lining and sublining compartments

PRG4-associated fibroblasts occupy the surface lining, whereas deeper fibroblasts include populations expressing thymocyte differentiation antigen 1 (THY1/CD90). Lineage studies and single-cell maps distinguish these compartments, although marker thresholds vary (14, 15). Experimental evidence for macrophage support of joint homeostasis comes largely from inflammatory arthritis. In the mouse model studied by Huang and colleagues, resident macrophage loss preceded arthritis; entry of competent monocytes restored the resident compartment and suppressed disease (16). Separate fate-mapping and imaging experiments identified a macrophage barrier around the joint (17). Whether an equivalent barrier protects human OA synovium remains unresolved.

Lining cells can remain present while surface function deteriorates. Mast-cell tryptase cleaved PRG4 and reduced its lubricating activity in tribological assays (18). In collagenase-induced OA (CiOA), C-X3-C motif chemokine receptor 1 (CX3CR1)-lineage macrophages shifted from the lining toward vascularized fibrotic regions (11). This redistribution shows why a resident-cell marker alone is insufficient to assign a protective function.

### 2.2 Disease-associated fibroblast and macrophage states

In human hip OA, epiregulin (EREG)-positive lining fibroblasts express inflammatory and matrix-remodeling programs (19). Pain-associated fibroblasts have been identified in mapped regions of knee synovium (20). Separate analyses of the infrapatellar fat pad-synovium unit identified biglycan-positive intermediate fibroblasts (21). CD34-high fibroblasts characterize another fibrotic knee phenotype (22). These descriptions come from different anatomical samples and comparison groups, which need to be considered when relating the populations to one another.

Profiling of human knee synovium also identifies inflammatory and regulatory macrophage programs (8, 23). A multi-tissue atlas reports more pain-marker-enriched macrophages and fewer regenerative stromal cells (24). In experimental post-traumatic OA (PTOA), recruited and resident-like myeloid populations change over time (25). Prolonged S100A8/A9 exposure elicits macrophage responses that do not fit a simple inflammatory-versus-reparative division (26). Shared markers therefore do not make clusters equivalent across studies. Expression of MER receptor tyrosine kinase (MERTK), lymphatic vessel endothelial hyaluronan receptor 1 (LYVE1), or collagen type I α1 chain (COL1A1) describes a cell state. Cell origin and function require additional evidence (17, 19).

Cell heterogeneity is easier to interpret when anatomical compartment, marker-defined state, and measured function are reported separately. PRG4 and THY1 help distinguish lining from sublining fibroblasts, whereas inflammatory, pain-associated, and matrix-producing labels describe programs that may overlap within these compartments (12, 15). Macrophages can be described in the same way by location and activation state. Resident or recruited origin should be assigned only when supported by the study design. This distinction permits comparison across studies without treating each cluster label as a stable cell type.

### 2.3 What spatial sampling adds

The pain-site study compared painful and non-painful regions within early OA knees. Its single-cell arm included paired painful and non-painful sites from four early OA donors and painful sites from four end-stage OA donors. Fibroblasts were sequenced after short outgrowth culture, so the resulting map reflects retained regional programs rather than the full cellular composition of the original tissue (20). Conditioned medium from early OA pain-site fibroblasts increased survival and neurite growth in adult rat dorsal-root-ganglion cultures over 24 hours. The mediators responsible remain unclear: a small paired protein-array comparison did not identify a single significantly different factor (20).

Another study analyzed synovial biopsies from 122 patients with advanced knee OA and selected 32 for spatial profiling (27). Groups were defined by pain severity and perivascular edema, with matching for sex, body mass index, and radiographic grade. All samples came from the lateral suprapatellar recess. In a functional subset of eight patients per group, the macrophage bead-phagocytosis index was 19.42 percentage points lower in the more painful group (27). The study identifies clinically relevant regions, although pain-related differences cannot be separated from the accompanying vascular phenotype. Within-joint, site-matched sampling would help determine whether these differences reflect regional conditions or a more general OA cell subtype. **Figure 1** outlines the niche model. **Table 1** summarizes the interaction evidence, and **Table 2** maps the cell states, markers, locations, functions, and study settings discussed in this review.

**Figure 1:**
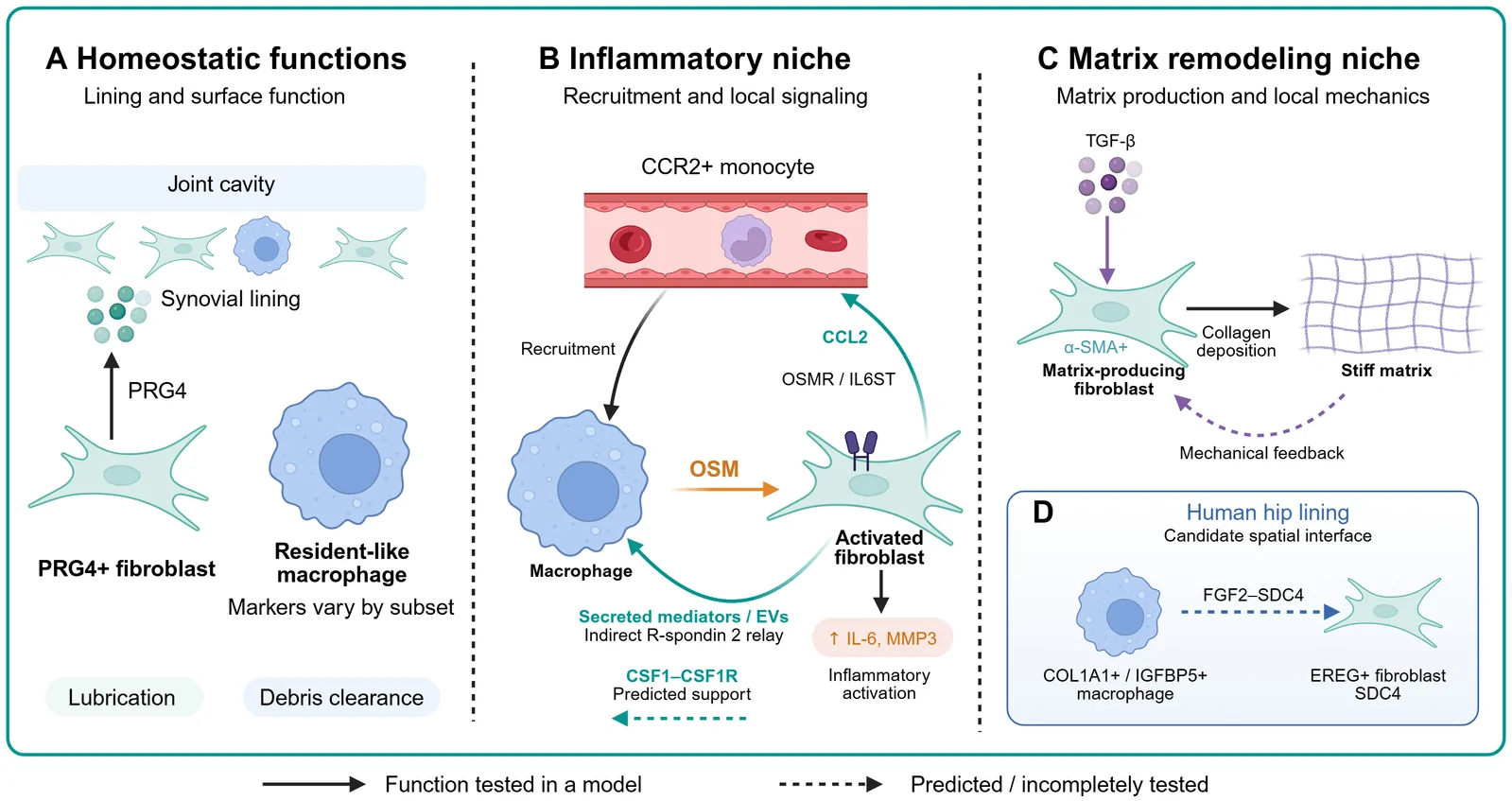
Spatial macrophage-fibroblast niches in osteoarthritis synovium. Homeostatic functions **(A)**, inflammatory signaling **(B)**, matrix remodeling **(C)**, and the human hip lining interface **(D)** represent potentially coexisting niches. Solid arrows indicate functionally tested effects; dashed arrows indicate candidate interactions. Evidence comes from different studies and does not establish a complete local circuit or an inflammation-to-fibrosis sequence. EVs, extracellular vesicles. Cell and collagen icons adapted from Servier Medical Art via Bioicons (CC BY 3.0).

**Table 1:** Evidence for synovial macrophage-fibroblast crosstalk.

Setting	Interaction or observation	Study evidence	Unresolved step
Homeostatic lining	PRG4-associated fibroblasts support lubrication; resident-like macrophages contribute to tissue maintenance	Mouse lubricin loss-of-function; physical macrophage barrier demonstrated in inflammatory arthritis (1, 17)	Equivalent barrier function in human OA
Recruitment and persistence	CCL2-CCR2 recruits monocytes; stromal CSF1/IL34 may support myeloid cells	CCL2/CCR2 perturbation in DMM; CSF1R communication predicted after ACL rupture (28, 29)	Required stromal source; functional test of CSF1/IL34
Macrophage-to-fibroblast activation	OSM increases inflammatory and catabolic genes	CiOA fate mapping and OSM challenge; migration and collagen were unchanged (11)	Macrophage-specific necessity and human tissue response
Fibroblast feedback	R-spondin 2 alters the fibroblast secretome; vesicles induce macrophage glycolysis	Conditioned-medium relay (12); mainly murine vesicle experiments implicating HIF-1α (30)	Secondary mediator, vesicle cargo, and transfer site in patients
Human knee phenotypes	Pain-associated vascular changes; CD34-high/CD70-high fibroblasts support immune regulation	Spatial profiling; CD70 blockade in tissue coculture (22, 27)	Whether phenotype-directed intervention preserves protective functions
Human hip lining interface	EREG-positive fibroblasts neighbor COL1A1/IGFBP5-positive macrophages	Single-cell/spatial profiling and immunofluorescence in FAI-associated hip OA (19)	Causal FGF2-SDC4 signaling and generalizability to knee OA

Evidence levels and study settings differ across rows. The CSF1 and R-spondin reports share a single-cell dataset.

**Table 2:** Cell states in synovial macrophage-fibroblast niches.

Cell state	Representative markers	Niche	Function and interpretation	Setting and source
Lining fibroblasts	PRG4-high; THY1-low/negative	Surface lining	Lubricant production; after injury, a PRG4-high subset secretes R-spondin 2 and can relay inflammatory signals.	Mouse lineage and PTOA studies (12, 15)
Sublining fibroblasts	THY1/CD90; CD34 varies	Sublining, including perivascular tissue	Connective-tissue and progenitor programs; anatomical compartment alone does not establish inflammatory function.	Mouse mapping; human knee OA (8, 15)
Inflammatory lining fibroblasts	EREG; PRG4-high; CCL20; MMP3-high	Hip synovial lining	Inflammatory and catabolic transcripts; proximity to matrix-associated macrophages suggests potential local interactions.	Human FAI-associated hip OA; spatial association (19)
Pain-associated fibroblasts	Early pain-site signature includes CXCL2, IL6 and LIF	Pain-mapped knee synovium	Conditioned medium supports neuronal survival and neurite growth; signature depends on disease stage and culture.	Human knee OA; rat neuronal assay (20)
Intermediate fibroblasts	BGN (biglycan)	Infrapatellar fat pad–synovium unit	Inflammatory and matrix-related programs; implicated with macrophages in cartilage-directed APOE signaling.	Human knee atlas; functional APOE studies (21)
CD34-high fibroblasts	CD34-high; CD70-high subset	Predominantly perivascular	Fibrosis-associated state with immune regulation: CD70 supports regulatory T cells; tissue blockade increases inflammatory myeloid responses.	Human knee OA; coculture perturbation (22)
Matrix-producing fibroblasts	COL1A1; ACTA2/α-SMA in contractile states	Matrix-rich stroma	Matrix synthesis and myofibroblast-like contractility; stiffness can impair lubricin output. Markers describe overlapping programs.	Human and mouse OA; bovine mechanobiology (6, 31)
Resident/resident-like macrophages	Mouse: CX3CR1 or TIMD4, LYVE1, FOLR2 programs	Lining and interstitial niches, depending on subset	Homeostatic/barrier functions in arthritis models; position and activation can change after injury.	Mouse arthritis and PTOA; lineage evidence differs (17, 29)
Recruited, OSM-associated macrophages	CCR2; human MERTK-low, CD48-high; mouse TIMD4-negative	Inflamed synovium; near vascularized fibrotic regions in CiOA	OSM-associated inflammatory signaling to fibroblasts; monocytic origin tested in mice.	Mouse CiOA fate mapping; human knee profiling (11)
Inflammatory/regulatory macrophage programs	MERTK-low, CD206-low versus MERTK-high, CD206-high	Human knee synovium; compartments vary	Low-marker state enriched in the inflammatory group; marker expression alone does not establish clearance function.	Human knee OA transcriptomic and tissue profiling (8, 22)
Pain-marker-enriched macrophages	P2RX7; inflammatory IL1B, IL6, NOS2 and TNF programs	Knee multi-tissue atlas; precise synovial niche unresolved	Transcriptomic association with inflammatory and pain-related programs; a direct contribution to pain has not been tested.	Human knee OA atlas (24)
Matrix-associated macrophages	CD68; COL1A1; IGFBP5	Hip lining near EREG-positive fibroblasts	Matrix-associated signature; candidate FGF2-SDC4 communication. Collagen deposition by these cells remains unproven.	Human FAI-associated hip OA; spatial association (19)

Cell-state descriptions may overlap. High/low and positive/negative indicate relative expression within each study, not common thresholds. Species and disease settings are not interchangeable. LIF, leukemia inhibitory factor; TIMD4, T-cell immunoglobulin and mucin domain containing 4; FOLR2, folate receptor β; P2RX7, P2X purinoceptor 7.

## 3 Macrophage-fibroblast crosstalk in inflammatory niches

### 3.1 Monocyte recruitment and stromal support

C-C motif chemokine ligand 2 (CCL2) recruits C-C motif chemokine receptor 2 (CCR2)-positive monocytes through a pathway supported by functional experiments. Human knee OA fibroblasts increased CCL2 secretion in response to cartilage debris or S100A8 (28). After destabilization of the medial meniscus (DMM) in mice, deletion of Ccl2 or Ccr2 reduced macrophage accumulation and cartilage damage, whereas deletion of Ccl5 or Ccr5 did not (28). The latter comparison indicates pathway selectivity. Oral bindarit or the CCR2 antagonist RS504393 also reduced damage during a 12-week postsurgical regimen (28). These experiments support blockade after injury, but leave open whether it can reverse established human OA and whether fibroblasts are the required tissue source of CCL2.

Fibroblasts may help sustain local macrophages through colony-stimulating factor 1 (CSF1). At days 7 and 28 after non-invasive anterior cruciate ligament (ACL) rupture, fibroblasts mainly expressed Csf1 and pericytes expressed Il34. Communication analysis predicted signaling to Csf1r-positive immune cells (29). This analysis reused the single-cell dataset from the R-spondin study discussed below (12, 29). It identifies a possible stromal survival signal whose functional importance remains to be tested.

The order of cytokine exposure can change fibroblast responses. In fibroblasts obtained during ACL reconstruction, interleukin-1β (IL-1β) induced chemokines, whereas subsequent transforming growth factor-β1 (TGF-β1) treatment reduced that response (32). The ACL-reconstruction cohort differs from an OA arthroplasty cohort; the response also argues against treating TGF-β1 as uniformly proinflammatory.

### 3.2 Macrophage signals that activate fibroblasts

Laouteouet and colleagues combined CiOA fate mapping with human macrophage sequencing from four OA donors (11). In CiOA mice, recruited macrophages expressed oncostatin M (OSM), and fibroblasts expressed its receptor components, OSM receptor (OSMR) and interleukin-6 signal transducer (IL6ST). Recombinant OSM increased fibroblast expression of Ccl2, Il6, and the matrix metalloproteinase genes Mmp3 and Mmp13. Migration in the Boyden-chamber assay was unchanged, as were collagen-I and fibronectin transcripts. Knee OSM challenge produced cartilage defects after three weeks without increasing tissue collagen (11). These results support inflammatory and catabolic amplification, but do not resolve whether OSM contributes to fibrosis. Independent network analysis also identified OSM as a candidate signal without establishing its cellular source (33).

Depletion experiments in human OA synovial cultures help quantify the macrophage contribution to inflammatory secretion. Macrophages comprised only 2-7% of dissociated cells, yet removal of CD14-positive cells reduced interleukin-6 (IL-6), interleukin-8 (IL-8), and CCL2 release by 70-80% (34). Combined tumor necrosis factor (TNF) and IL-1 inhibition had additive effects, indicating partially redundant inputs (34). Chondrocyte-conditioned medium also increased IL-6 release from OA synovial fibroblasts (35). Cytokine combinations increased fibroblast expression of aggrecanase-1 (ADAMTS4) (36). Multiple inputs may therefore sustain fibroblast activation when a single mediator is blocked.

In these human cultures, macrophages contribute to inflammatory secretion despite their small proportion (34). Alongside the OSM experiments, these findings show how macrophage signals can amplify stromal inflammation. The contribution of an individual mediator will depend on the local cell mixture and stimulus (11, 34).

Changing macrophage signaling and depleting macrophages can have different tissue effects. Macrophage-targeted signal transducer and activator of transcription 6 (STAT6) inhibition reduced mechanical pain sensitivity and synovitis in experimental rat OA without worsening tissue pathology. Macrophage depletion also reduced pain, but increased synovial fibrosis and vascularization (37). In human OA fibroblasts and explants, conditioned medium containing growth arrest-specific 6 (Gas6) suppressed inflammatory responses, and Axl inhibition abolished the effect (38).

### 3.3 Fibroblast feedback to macrophages

R-spondin 2 can alter macrophage responses indirectly by changing what fibroblasts secrete. In ACL-rupture PTOA, Rspo2 was enriched in PRG4-high lining fibroblasts (12). The tested macrophages lacked leucine-rich repeat-containing G protein-coupled (Lgr) receptors and did not respond directly to R-spondin 2. Conditioned medium collected after the treated fibroblasts were washed nevertheless altered macrophage inflammatory gene expression (12). The response therefore involves an indirect stromal relay, although the active secondary mediator has not been identified.

Extracellular vesicles provide another route for fibroblast-to-macrophage signaling. Exosomes from IL-1β-stimulated mouse fibroblasts increased hypoxia-inducible factor 1α (HIF-1α)-dependent glycolysis in RAW264.7 macrophages. Hif1a knockdown and PX-478 weakened this response, while vesicle injection aggravated DMM pathology (30). Human OA fibroblast vesicles also activated THP-1-derived macrophages (30). The glycolytic mechanism was tested mainly in murine cells; the human experiments used a cell line, leaving its relevance to primary patient macrophages unresolved. A separate diabetic OA study linked fibroblast Yes-associated protein 1 (YAP1)-regulated glycolysis to macrophage infiltration (39), but that association needs to be interpreted within its metabolic setting.

Macrophages and fibroblasts can therefore reinforce each other’s inflammatory activity. Fibroblast chemokines recruit monocytes, while macrophage signals increase fibroblast inflammatory and catabolic output (11, 28). Fibroblast-conditioned medium and extracellular vesicles can, in turn, alter macrophage activation (12, 30). These experimentally tested components suggest a local positive-feedback circuit, although the complete circuit has not been traced within one OA joint. CSF1-dependent macrophage persistence remains a candidate component, with no functional evidence yet that it is required for this circuit (29).

### 3.4 Consequences of inflammatory activation

Across these studies, the clearest effect of crosstalk is increased inflammatory activity, linking chemokine production to immune recruitment and catabolic responses. Fibroblast activation does not necessarily culminate in fibrosis: OSM induces inflammatory and cartilage-degrading programs without increasing collagen deposition (11). Pain, synovitis, and matrix accumulation therefore need to be measured separately. Keeping these outcomes distinct also allows inflammatory and fibrotic niches to be compared without assuming a temporal sequence.

## 4 Fibrotic niches and their relationship to inflammation

### 4.1 Clearance and persistence

Failure to clear dying cells may prolong inflammation. In a human knee study, CD14-positive synovial macrophages were challenged with apoptotic Jurkat cells. Late-OA macrophages showed reduced efferocytosis, the engulfment of apoptotic cells. OA synovial fluid also impaired clearance by blood-derived macrophages, with the strongest inhibition caused by fluid from highly painful, inflamed early-OA knees (40). The defect may arise from the local fluid environment as well as macrophage state and should not be attributed solely to late disease. Reduced bead uptake also accompanied worse pain and vascular abnormalities in the spatial knee cohort (27). Efferocytosis and bead uptake measure different forms of clearance; neither assay establishes when dysfunction begins.

Impaired clearance may sustain local inflammatory stimulation, whereas fibrosis must be assessed through matrix production and organization (6, 40). Human studies describe inflammatory and fibrotic features cross-sectionally (19, 22). Experimental injury datasets resolve changes over days 7 and 28 (12, 29). These designs capture tissue states in different contexts, rather than a continuous course from inflammation to fibrosis. Longitudinal and perturbation studies are needed to determine whether persistent inflammation initiates or maintains fibrotic niches, or whether mechanical and stromal programs develop partly independently.

### 4.2 Matrix production and mechanical responses

TGF-β-responsive matrix genes are increased in human OA fibroblasts, experimental OA synovium, and end-stage patient tissue. Remst and colleagues found shared increases in COL1A1, COL5A1, PLOD2, LOX, and TIMP1 (6). Cross-linking enzymes were among these genes, consistent with a contribution of matrix organization, as well as synthesis, to collagen persistence (6). Nuclear protein 1 (NUPR1) knockdown or treatment with trifluoperazine reduced synovial fibrosis in DMM mice (41).

The mechanobiology study compared human synovium from four asymptomatic donors and eight arthroplasty patients, while using juvenile bovine cells for its primary fibroblast experiments (31). Cells formed more contractile stress fibers on 55-kPa than on 5-kPa substrates. TGF-β increased lubricin secretion, but substrate stiffness blunted the response. Both fasudil and verteporfin reduced α-smooth muscle actin (α-SMA), whereas only fasudil partly restored PRG4 output and improved conditioned-medium lubrication (31). The difference illustrates why reducing a fibrosis marker does not necessarily restore tissue function. Whether aged human fibroblasts respond similarly to these mechanical perturbations remains unknown.

Preclinical studies also implicate Piezo1 in the response to mechanical overload (42). Fibroblast growth factor 10 (FGF10) reduced synovial fibrosis by inhibiting IL-6/Janus kinase 2 (JAK2)/signal transducer and activator of transcription 3 (STAT3) signaling in experimental models (43). Localization of platelet-derived growth factor (PDGF)-C/D in human tissue identifies possible local growth signals (44). PDGF receptor β (PDGFRβ)-lineage tracing indicates that perivascular cells contribute to myofibroblasts after injury (45). Two weeks after DMM, labeled cells in the infrapatellar fat pad were found away from vessels and expressed α-SMA and extra domain A (ED-A) fibronectin. By eight weeks, the labeled population had shifted toward a perivascular distribution (45). The distribution therefore changes over time, although the role of individual PDGF ligands remains unresolved.

In human OA fibroblasts, 15-deoxy-Δ12,14-prostaglandin J2 enhanced prednisolone suppression of TGF-β-induced fibrotic responses (46). Evidence from RA fibroblasts and lung fibrosis supports testing YAP1 and the transcriptional coactivator with PDZ-binding motif (TAZ) in OA (47, 48). PRG4 reduced myofibroblast features in cultured OA synoviocytes, partly through CD44. PRG4 re-expression also reduced fibrosis markers in Cd44-deficient mice (49), indicating that some reported PRG4 effects do not require CD44. Measuring deposited matrix and lubrication alongside α-SMA would distinguish changes in marker expression from changes in synovial function.

### 4.3 The human hip inflammatory-fibrotic interface

The hip study compared six patients with femoroacetabular impingement (FAI) and six with OA secondary to FAI. Spatial transcriptomics included four FAI and six OA samples (19). EREG-positive lining fibroblasts were located near CD68-positive macrophages expressing COL1A1 and insulin-like growth factor-binding protein 5 (IGFBP5). Immunofluorescence quantified nearby cell pairs, and communication analysis identified fibroblast growth factor 2 (FGF2)-syndecan 4 (SDC4) as a candidate signal between these populations (19). The fibroblasts had an inflammatory lining identity; they had not been established as deep-tissue myofibroblasts.

This arrangement brings inflammatory fibroblasts into proximity with macrophages expressing a matrix-associated transcriptional program (19). It identifies an anatomical site where the programs could interact without either population becoming a myofibroblast. The small cohort and FAI comparator limit generalization. Blocking FGF2-SDC4 signaling in mapped regions would test the predicted interaction. Assigning collagen deposition to COL1A1-expressing macrophages would require a separate matrix-production assay.

At the knee fat pad-synovium interface, intermediate fibroblasts and macrophages were implicated in apolipoprotein E (APOE) signaling (21). Soluble APOE accelerated proteoglycan loss from human cartilage, and local neutralization attenuated CiOA (21). These experiments establish an effect on cartilage; whether the signal also mediates reciprocal induction between the two synovial cell populations remains untested.

### 4.4 Fibrosis does not define a uniform harmful state

The CD34-high fibroblast study illustrates why fibrosis alone is an inadequate basis for stromal depletion. Patients in its fibrotic knee group reported less pain than those in its inflammatory group, and CD34-high/CD70-high fibroblasts occupied perivascular regions. CD70 blockade in synovium-cartilage coculture reduced the proportion of regulatory T cells and increased the proportion of IL-1β-positive macrophages. A complementary CD70-overexpression experiment used the MRC-5 fibroblast line rather than primary OA fibroblasts (22). The findings support immune regulation by CD70-associated stroma, although this role cannot be assigned to all matrix-producing cells. Neural and vascular changes also contribute to pain beyond collagen burden (50). Pain relief, matrix remodeling, and preservation of immune regulation should therefore be measured as separate outcomes.

Matrix remodeling and immune regulation are distinct aspects of stromal function. Fibrotic regions may contain both cells that restrain inflammation and cells that alter tissue mechanics (22, 31). Their overall effect depends on the cells present and what they produce, so collagen abundance or a single fibroblast marker is unlikely to capture the function of the niche.

## 5 Therapeutic and methodological implications

A priority is to test whether macrophage-derived OSM is required for the responses observed in gain-of-function experiments, given the likely macrophage source (11). Macrophage-specific Osm perturbation should be paired with measurements of fibroblast inflammatory output and matrix production. Stromal Csf1 deletion would test whether the predicted support signal is needed for local myeloid persistence (29). Both approaches remain research priorities; their therapeutic value requires further study.

Human validation needs to preserve anatomical context. Sampling adjacent inflamed and collagen-rich regions within the same joint would reduce some between-patient variation. Pain location, radiographic grade, and vascular edema should be recorded to interpret the comparison. Donors should remain the independent units for inference, rather than cells or imaging regions (51). Comparisons of early and end-stage samples can identify differences, but inferring progression requires repeated or longitudinal sampling.

Functional studies should assess preservation of synovial function as well as disease-associated changes. Efferocytosis and PRG4-dependent lubrication need to be measured alongside inflammatory mediators and collagen deposition. The macrophage-depletion experiments and CD70 blockade caution against indiscriminate removal of synovial cells or inhibition of their signals (9, 22). Local delivery and short treatment windows warrant testing to determine whether they improve outcomes. Barrier studies in mouse inflammatory arthritis can inform assay design, although they do not establish efficacy in OA (17).

## 6 Discussion and conclusion

Experimental evidence supports a contribution of macrophage-fibroblast crosstalk to synovial inflammatory amplification. Macrophage depletion and cytokine perturbation show that myeloid signals influence inflammatory output (11, 34). Fibroblast secretions can, in turn, alter macrophage recruitment and activation (28, 30). Spatial studies identify anatomical sites where these interactions may occur (19, 27). The evidence favors interacting, potentially coexisting niches whose effects depend on cell state and location. It does not establish a single course from inflammation to fibrosis.

Inflammatory activation, matrix remodeling, and tissue maintenance are partly separable within this model. Matrix-associated macrophage signatures do not establish collagen deposition, and fibrosis-associated fibroblasts can also support immune regulation (19, 22). The therapeutic question is therefore which local interactions can be modified while preserving clearance and lubrication. Combining cell-specific perturbation with spatially matched human tissue would help separate shared inflammatory circuits from niche-specific mechanisms and clarify their contributions to pain and structural disease.
